# Advanced Multifunctional Coatings for New Applications: A New, Open Special Issue of *Materials*

**DOI:** 10.3390/ma18225250

**Published:** 2025-11-20

**Authors:** Tao Wang, Stephan Handschuh-Wang

**Affiliations:** 1 School of Integrated Circuits, Shenzhen Polytechnic University, Shenzhen 518055, China; 2 Advanced Energy Storage Technology Center, Shenzhen Institutes of Advanced Technology, Chinese Academy of Sciences, Shenzhen 518055, China; 3 College of New Materials and New Energies, Shenzhen Technology University, Shenzhen 518118, China; wangstephan@sztu.edu.cn

From information technology, life, and healthcare to the energy revolution and sustainable development, a series of major technological breakthroughs depend on the substantial improvement of material properties. Coatings have long played an important role in the development and optimization of materials and devices. Coatings are often used to protect delicate objects; for instance, objective lenses are coated to protect against scratches, and architectural metalwork is coated with lacquer or hard metal coatings to protect against corrosion and wear. In our daily lives, we are surrounded by objects and devices with multifunctionality, a trait that grants them added value and good value prospects. One area in which multifunctionality is encountered is the smartphone; it can be used to call somebody, but it can also be used to check E-mails, play music like an MP3-player, surf websites, and play games. Similarly, a smartphone’s display allows the user to control the phone by touch and displays images in high fidelity, while only two decades ago, such screens were only capable of displaying crude images. Akin to bundling functionality in electronic devices, multifunctional coatings have emerged. These coatings not only provide the surface with one basic property but also add new functions (e.g., sensing or bactericidal activity). 

This Special Issue presents novel scientific and engineering research, making contributions to our discovery and understanding of advanced multifunctional coatings, novel synthesis technology, growth mechanisms, characterization, and advanced coating applications. Multifunctional coatings can be made of a variety of materials and structures, and the studies herein cover different coating systems, such as diamond, polymer, ceramic, metal, and metal oxide coatings. In a work published in this Special Issue, Araiza-Ibarra et al. studied the deposition of HfO_2_ on sapphire and silicon via magneton sputtering [1], highlighting the effect of sputtering power density on the morphology of the coating and investigating the dewetting and buckling of the coating at high deposition power. Buckling and dewetting are not only deleterious for the optical properties but lead to film delamination. HfO_2_ films have been applied in resistive switching and ferroelectric memories [2]. In this case, the buckling and dewetting is likely linked to poor film adhesion/bonding and large compressive residual stress.

Indeed, large stresses (intrinsic and thermal) are known to be detrimental to film adhesion. For instance, in diamond coatings grown on most substrates, great compressive stress can be observed, stemming from a mismatch of the coefficient of thermal expansion between diamond and substrates. To avoid great compressive stresses and tool failure, Zhou et al. optimized the pre-treatment (etching) of the microdrills and used a diamond/SiC composite interlayer prior to diamond deposition [3]. This reduced the compressive stress of the diamond coating and bonding of the coating to the microdrills and improved tool lifetime, drilling performance, and hole quality. 

Xia et al. sought to protect aluminum from corrosion by coating it with a superhydrophobic polymer coating [4]. A mixture of hexadecyltrimethoxysilane (HDTMS)-modified SiO_2_ nanoparticles and acid-catalyzed silica sols with polydimethylsiloxane (PDMS) binder was used and sprayed onto aluminum. The silica nanoparticles in the binder formed a network imparting both wear resistance and roughness, enabling superhydrophobicity. A water impact test showed good bonding, wear resistance, and corrosion resistance, the last of which was perhaps related to the hydrophobic recovery of PDMS [5].

The studies published in this Special Issue point to a future where coating systems will be stronger and resistance to wear will be greater. At the same time, new functionalities will be employed, making coatings smarter and adaptive.
